# Intraperitoneal Delivery of Iopamidol to Assess Extracellular pH of Orthotopic Pancreatic Tumor Model by CEST-MRI

**DOI:** 10.1155/2023/1944970

**Published:** 2023-01-05

**Authors:** Bruna Victorasso Jardim-Perassi, Pietro Irrera, Justin Y. C. Lau, Mikalai Budzevich, Christopher J. Whelan, Dominique Abrahams, Epifanio Ruiz, Arig Ibrahim-Hashim, Sultan Damgaci Erturk, Dario Livio Longo, Shari A. Pilon-Thomas, Robert J. Gillies

**Affiliations:** ^1^Department of Cancer Physiology, H. Lee Moffitt Cancer Center and Research Institute, Tampa, FL, USA; ^2^Small Animal Imaging Laboratory, H. Lee Moffitt Cancer Center and Research Institute, Tampa, FL, USA; ^3^Department of Biological Sciences, University of Illinois, Chicago, IL, USA; ^4^Comparative Medicine, University of South Florida, Tampa, FL, USA; ^5^Institute of Biostructures and Bioimages (IBB), National Research Council of Italy (CNR), Turin, Italy; ^6^Department of Immunology, H. Lee Moffitt Cancer Center and Research Institute, Tampa, FL, USA

## Abstract

The extracellular pH (pHe) of solid tumors is often acidic, as a consequence of the Warburg effect, and an altered metabolic state is often associated with malignancy. It has been shown that acidosis can promote tumor progression; thus, many therapeutic strategies have been adopted against tumor metabolism; one of these involves alkalinization therapies to raise tumor pH to inhibit tumor progression, improve immune surveillance, and overcome resistance to chemotherapies. Chemical exchange saturation transfer-magnetic resonance imaging (CEST-MRI) is a noninvasive technique that can measure pH *in vivo* using pH-sensitive contrast agents. Iopamidol, an iodinated contrast agent, clinically used for computed tomography (CT), contains amide group protons with pH-dependent exchange rates that can reveal the pHe of the tumor microenvironment. In this study, we optimized intraperitoneal (IP) delivery of iopamidol to facilitate longitudinal assessments of orthotopic pancreatic tumor pHe by CEST-MRI. Following IV-infusion and IP-bolus injections, we compared the two protocols for assessing tumor pH. Time-resolved CT imaging was used to evaluate the uptake of iopamidol in the tumor, revealing that IP-bolus delivered a high amount of contrast agent 40 min postinjection, which was similar to the amounts reached with the IV-infusion protocol. As expected, both IP and IV injection protocols produced comparable measurements of tumor pHe, showing no statistically significant difference between groups (*p*=0.16). In addition, we showed the ability to conduct longitudinal monitoring of tumor pHe using CEST-MRI with the IP injection protocol, revealing a statistically significant increase in tumor pHe following bicarbonate administration (*p* < 0.001). In conclusion, this study shows the capability to measure pHe using an IP delivery of iopamidol into orthotopic pancreatic tumors, which is important to conduct longitudinal studies.

## 1. Introduction

The Warburg effect is a hallmark of cancer that refers to the tendency of cancer cells to exhibit the high activity of glycolysis and low activity of mitochondrial oxidative phosphorylation, regardless of oxygen availability [1]. As a consequence of this metabolic shift in cellular energy production in cancer cells, there is an overproduction of lactic acid resulting in a decrease of the extracellular pH (pHe), which is often associated with malignancy and resistance to therapies [2, 3]. Thus, neutralization of tumor acidosis is a therapeutic strategy that has been explored to inhibit invasion and metastasis, improve immune surveillance, and overcome resistance to therapy [4, 5].

In this context, assessment of tumor pHe *in vivo* can improve diagnoses, inform treatment decisions, and help in evaluating responses to therapies when longitudinal measurements can be implemented [6]. Several noninvasive methods have been developed to monitor tumor pH *in vivo*, such as optical imaging, positron emission tomography (PET), electron paramagnetic resonance (EPR) spectroscopy, MR spectroscopy, as well as MRI with relaxivity-based contrast agents [7, 8]. However, these techniques can be inaccurate, insensitive, or technically challenging to implement in the clinic [8].

Chemical exchange saturation transfer-magnetic resonance imaging (CEST-MRI) is an emerging noninvasive technique that can accurately measure pHe *in vivo* and create spatial pHe maps [9–11]. Iodinated contrast agents, such as iopamidol, can be used as it contains two chemically different amide protons that resonate at distinct frequencies and can exchange with the protons of the bulk water in a pH-dependent manner, providing a basis for the establishment of the ratiometric approach to determine pH [12, 13]. Those amide protons resonate at frequencies of 4.2–5.5 ppm from water, and the ratio of two CEST effects can be used to measure pH in a manner that is independent of concentration, endogenous T1 relaxation time, and incomplete saturation [14]. The first demonstration of imaging pH using iopamidol and similar agents was performed in the kidneys of mice in 2011 [15] and later it was also demonstrated in tumors [16]. More recently, pH imaging with iodinated agents has been successfully demonstrated in humans [17–19].

Iopamidol is a clinically approved computed tomography (CT) contrast agent and can be administered intravenously (IV) safely. In preclinical studies with mice, IV injections are commonly performed to deliver contrast agents for MR imaging [14, 20]. However, tail vein IV injection requires technical expertise and skill, while intraperitoneal injection (IP) is technically simple and fast. More importantly, repeated tail vein IV catheterizations are challenging as they can cause venous thrombosis and wounds of the tail interfering with further injections for longitudinal studies [21].

Our aim is to test the feasibility of using IP injection to deliver iopamidol into orthotopic pancreatic tumors in order to accurately measure tumor pHe by CEST-MRI. We have especially focused on optimizing a CEST-MRI protocol with IP delivery of iopamidol to facilitate use in further studies to measure pHe longitudinally in response to IV-delivered alkalinization therapies.

## 2. Methods

### 2.1. Mouse Tumor Models

Murine pancreatic cancer cell lines UN-KPC-961 (KPC961) and Panc02 were used to develop orthotopic tumor models. The KPC961 pancreatic cell line was obtained via MTA from Dr. Batra (University of Nebraska Medical Center, Omaha, NE) [22], and Panc02 was a kind gift from Dr. Emmanuel Zervos (East Carolina University, Greenville, NC) [23].

KPC961 and Panc02 cells were tested negative for mycoplasma and were inoculated orthotopically in immunocompetent B6.129 and C57BL/6 mice.

An additional KPC961 clone 1B6 tumor model, which expresses a human cell surface protein CEACAM6 was used to test changes in pH following bicarbonate administration.

KPC961 parental cells were cultured in DMEM/F12 media (Gibco, Waltham, MA) and Panc02 cells in Roswell Park Memorial Institute (RPMI) 1,640 culture media (Gibco, Waltham, MA), both supplemented with 10% fetal bovine serum (FBS) and 1% of penicillin/streptomycin (P/S) (Sigma, St. Louis, MO) and maintained in an incubator at 37°C and 5% CO_2_. KPC961 clone 1B6 cells were cultured in DMEM/F12 media (Gibco, Waltham, MA) supplemented with 10% FBS, 1% of P/S (Sigma, St. Louis, MO), and 5 *µ*g/ml of puromycin (Gibco, Waltham, MA).

### 2.2. Orthotopic Pancreatic Tumor Inoculation

Animal experiments were approved by the Institutional Animal Care and Use Committee (IACUC) (Protocol #6834). Mice were obtained from Charles River Laboratory (Wilmington, MA) and housed in a facility under pathogen-free conditions in accordance with IACUC standards of care at the H. Lee Moffitt Cancer Center.

For tumor inoculation, cells were resuspended in cold phosphate-buffered saline (PBS) at a concentration of 5 × 10^4^ KPC961 cells and 1 × 10^4^ Panc02 cells in 20 *µ*l of sterile PBS. To provide analgesia, mice were dosed with Meloxicam (5 mg/kg) 30 min prior to surgery. Mice were anesthetized with 2% isoflurane delivered in 1.5 l/min oxygen ventilation and the hair was removed from the midsection of each mouse. The skin was sterilized with topical solutions. The abdominal skin and muscle were incised just off the midline and directly above the pancreas to allow visualization of the pancreatic lobes; the pancreas was gently retracted and positioned to allow for direct injection of a 20 *µ*l bolus of cells/PBS into the head of the pancreas. The pancreas was placed back within the abdominal cavity. Closure of the abdominal cavity was accomplished in two layers, with the application of an absorbable peritoneal layer in a continuous pattern, stitched closed with absorbable suture. Then, the skin layer was closed in a simple interrupted pattern with nonabsorbable surgical staples, which were removed 10 days postinoculation.

### 2.3. Tumor Visualization by Ultrasound Imaging

Starting 6 days of postsurgery, we used ultrasound imaging to confirm the establishment of pancreatic tumors and to measure the initial volume, in order to plan the CT and CEST experiments. Mice were imaged with Vevo 2100 ultrasound system (FUJIFILM VisualSonics Inc., Toronto, Canada). For tumor volume measurement, regions of interest (ROIs) were taken at parallel slices. Scans were done at 0.05 mm down to 0.01 mm thickness, and conducted with the 3D motor attachment. Mice were anesthetized with 1.5%–3% isoflurane delivered via nose-cone manifold, depilated with Nair, and positioned with surgical tape onto a thermoregulated stage where electrodes and rectal probe continuously monitor their body temperature, heart rate, and respiration rate. Prewarmed ultrasound gel and an adjustable heat lamp maintained animal warmth.

### 2.4. Iopamidol Injection Protocols

Iopamidol (Isovue-370; Bracco Diagnostics) was used as a contrast agent for CT imaging and CEST-MRI. Two injection protocols were tested to administer iopamidol into pancreatic orthotopic tumors either through IV or IP routes.

**Figure 1:**
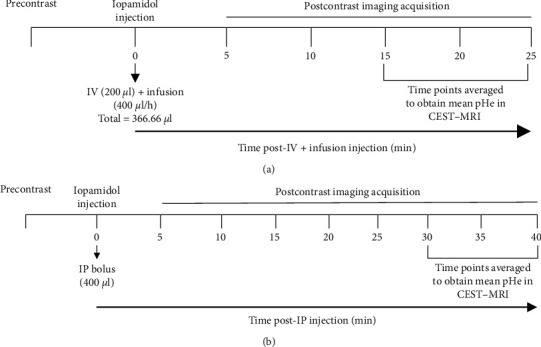
CT and CEST-MRI imaging flowchart. For both CT and CEST-MRI, a precontrast imaging was acquired, followed by the injection of iopamidol and postcontrast imaging acquisition for 25 min for the IV + infusion injection protocol (a) and 40 min postcontrast for the IP injection protocol (b).

Mice underwent precontrast imaging followed by iopamidol injection and postcontrast imaging acquisition. IV + infusion injection protocol consisted of a bolus of 200 *µ*l of iopamidol administered through an IV polyurethane 26-gauge needle catheter (Covidien, Ireland) followed by infusion at 400 *µ*l/hr and postcontrast imaging acquisition for 25 min. IP bolus protocol consisted of 400 *µ*l of iopamidol administered through an IP polyurethane 26-gauge needle catheter (Covidien, Ireland) and postcontrast imaging acquisition for 40 min.  Figure 1 shows a flowchart with CT and MRI timelines and injection protocols.

### 2.5. Computed Tomography

*In vivo* CT imaging (pre- and postcontrast injection) was done with the IV + infusion protocol for the KPC961 tumor model and with the IP bolus injection protocol for the KPC961 and Panc02 tumor models.

For the IV + infusion protocol, data were acquired once before contrast and five times postcontrast (total of 25 min postcontrast imaging acquisition). For the IP injection protocol, CT imaging was acquired once before contrast and eight times postcontrast, for a total of 40 min postcontrast imaging acquisition (Figure 1). After establishing that the highest iopamidol uptake was at 40 min postcontrast with the IP injection in the KPC961 tumor model, subsequent CT scans were performed before contrast and only at 40 min postcontrast in additional mice of the KPC961 model as well as in a different orthotopic pancreatic tumor model (Panc02).

A Siemens Inveon PET/SPECT/CT scanner was used for all CT measurements. The mice were imaged in the prone position. All animals were anesthetized with 2%–4% isoflurane anesthesia before and during image acquisition. Thermally controlled stages were incorporated within the CT scanner setup to maintain normal body temperature. A step-and-shoot protocol [14] was employed with 300 projections through a 1.5 mm aluminum filter covering 220° of rotation arc with 410 ms exposure time per projection at 80 kVp and 500 mA. The total scan time was 346 s. The 4 × 4 binning option provided a 27.9 × 41.9 × 22.7 mm^3^ field of view. A Feldkamp filtered backprojection algorithm was used to reconstruct a stack of 416 slices of 512 × 512 pixels with an isotropic voxel size of 54.53 *μ*m.

Reconstructed CT images were analyzed with in-house-developed MATLAB code. 3D regions of interest (ROIs) were drawn around the tumors in the CT images. Precontrast images were used as a background. The precontrast mean intensity (in Hounsfield units (HU)) was subtracted from each postcontrast mean intensity to obtain the contrast-related change in CT number (*Δ*HU).

### 2.6. CEST-MRI

CEST-MRI was performed using the IV + infusion and IP injection protocols in the KPC961 orthotopic tumor model (*n* = 3 mice for each injection protocol). In addition, three mice with KPC961 clone 1B6 orthotopic tumors were used to evaluate the ability to perform longitudinal CEST-MRI with the IP protocol. For these experiments, CEST imaging was performed to obtain a baseline tumor pHe and 2 days later, mice were provided with 1 M bicarbonate by gavage (0.7 ml) [24] and CEST imaging started 45 min following gavage.

Before imaging acquisition mice were given intramuscular xylazine (6 mg/kg) as a muscle relaxant (AnaSed, Akorn Pharmaceutical, Illinois) and anesthetized with 2% isoflurane delivered in 1.5 l/min oxygen ventilation during MRI scanning. Respiratory function was maintained at a range of 60–80 breaths per minute and body temperature was maintained at 37°C by an external heater and continuously monitored using a rectal thermometer (SA Instruments Inc, System 1025, Stony Brook, NY).

Mice were imaged in a 7T horizontal-bore magnet (Agilent ASR 310, Santa Clara, CA; Bruker Biospin, Inc. BioSpec AV3HD, Billerica, MA), using a ^1^H 30 mm volume coil (m2m Imaging Corp, Cleveland, OH), following the protocol described in Longo et al. [20]. A single T_2_-weighted axial slice crossing the center of the tumors was acquired with TR = 2.3 s, TE = 30.8 ms, NA = 2, slice thickness = 1 mm, FOV = 35 × 35 mm, matrix size = 128 × 128, which yielded an in-plane resolution of 273 *μ*m. CEST images were acquired with presaturation using continuous wave (CW) RF irradiation (3 *μ*T for 5 s) at 46 frequency offsets unevenly distributed from −10 to 10 ppm relative to the water resonance followed by a single-shot RARE imaging sequence (TR = 6.0 s, effective TE = 5.83 ms, centric encoding, slice thickness = 1 mm, FOV = 35 mm, matrix size = 64 × 64, in-plane spatial resolution = 546 *μ*m, NA = 1), with the acquisition time for each Z-spectrum being 4 min 36 s. Z-spectra collection was performed once before contrast and repeated five times postcontrast when using the IV + infusion protocol and eight times postcontrast when using the IP injection protocol, totaling 23 and 36 min and 48 s postcontrast for each injection protocol, respectively.

CEST images were analyzed using MATLAB with a homemade script based on a multipool Lorentzian fitting to determine iopamidol contributions at 4.2 and 5.5 ppm [25]. Background subtraction was achieved using joint least-squares fitting of the precontrast and each postcontrast Z-spectrum to determine the contrast-related contribution to the CEST signal. pH values for each voxel were determined by interpolating the ratio between the two frequencies to a calibration curve obtained from *in vitro* experiments performed on the same MR system with 20 mM iopamidol solutions at different pH (5.5–7.9) (Supplementary Figure S1). To obtain a mean pHe for the tumor ROI of each mouse, we averaged the pHe values obtained in the last three postcontrast time points for each injection protocol (15–25 min for IV + infusion and 30–40 min for IP) (Figure 1), which is based on CT results presented similarly high amounts of iopamidol (*Δ*HU > 40).

### 2.7. Empirical Cumulative Distribution Functions of pHe

To compare the efficacy of injecting iopamidol by IP versus IV, we constructed empirical cumulative density functions (ECDF) of pHe pixel distributions in the tumor ROI of the six mice with KPC961 orthotopic tumors imaged with CEST-MRI (three mice administered with iopamidol by IP and three by IV plus infusion). We then combined pHe measurements for each of the three mice contingent upon IP versus IV administration obtained at the three last postcontrast time points, as described below in the results section (30, 35, and 40 min postcontrast for IP, and 15, 20, and 25 min postcontrast for IV plus infusion; Figure 1). We constructed the ECDF curves from the combined pHe measurements with package *ggplot2* using R programming language version 4.0.0 [26]. We then tested whether the ECDF curves differed significantly with a Kolmogorov–Smirnov test using R package *dgof*. We used the “jitter” command to eliminate tied pHe values (IP versus IV). Statistical significance was assessed at *p* ≤ 0.05.

To determine how pHe varied across the tumors in response to bicarbonate administration, we constructed ECDF of pHe pixel distributions in tumor ROIs of three mice with KPC961 clone 1B6 orthotopic tumors imaged with CEST-MRI before and after bicarbonate administered as described above. To establish baseline pHe of the tumor ROI, we combined all the pHe measurements by pixel for each mouse obtained in the last three postcontrast time points (30, 35, and 40 min postcontrast) following IP injection. We next combined all the pHe measurements by pixel from the same three mice at three time points (30, 35, and 40 min postcontrast; Figure 1) following bicarbonate gavage 2 days later. We constructed the ECDF curves pre- and postbicarbonate administration and tested whether they differed significantly using a Kolmogorov–Smirnov test, as described above.

## 3. Results

### 3.1. Iopamidol Uptake in Orthotopic Pancreatic Tumors Evaluated by CT

Time series CT was used to evaluate the uptake of iopamidol into orthotopic pancreatic tumors. Precontrast CT imaging was performed followed by iopamidol injection by either IV or IP routes and postcontrast CT imaging was performed for 25 or 40 min for each injection protocol, respectively (Figure 1).

**Figure 2:**
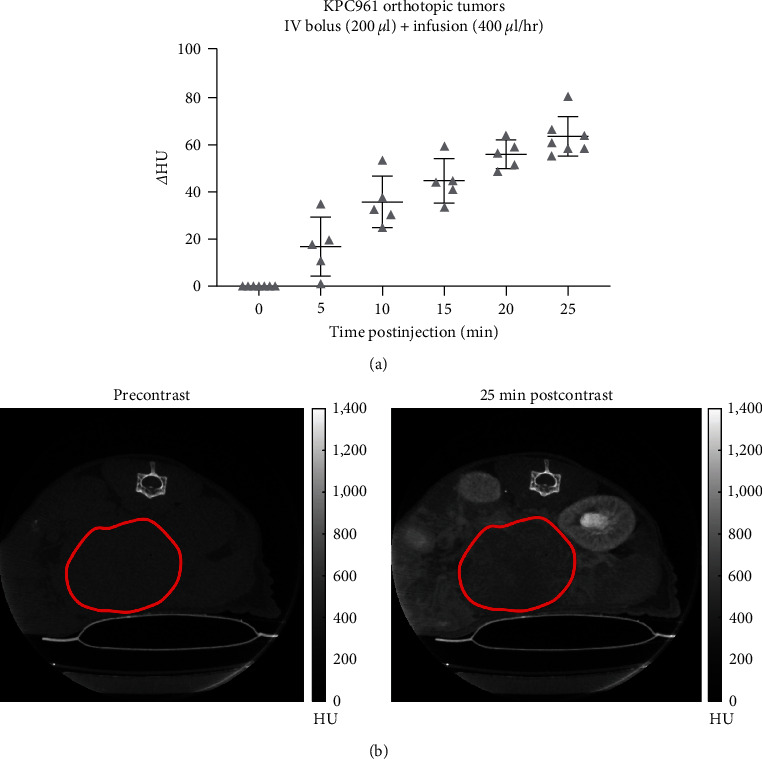
*In vivo* CT data showing the uptake of iopamidol over time into KPC961 orthotopic tumors following the IV + infusion protocol. (a) Delta Hounsfield units (*Δ*HU) was obtained by subtracting the mean intensity from the tumor ROI in each postcontrast CT image (5–25 min) from the mean intensity of the tumor ROI in the precontrast CT image; (b) CT images showing an axial view and the tumor ROI in red, before and 25 min postiopamidol administration using the IV + infusion protocol. Hounsfield units (HU).

**Figure 3:**
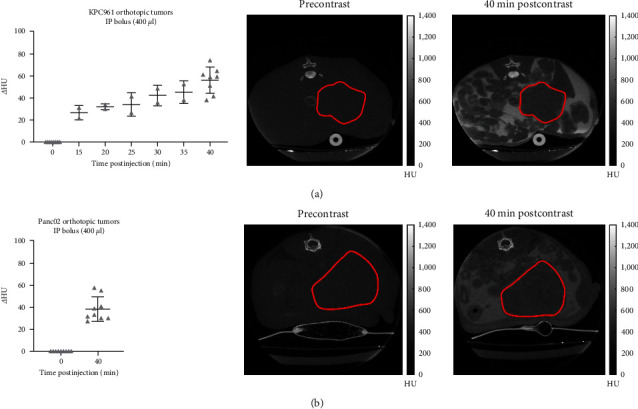
*In vivo* CT data showing the uptake of iopamidol into orthotopic pancreatic tumors following IP bolus injection. (a) Quantification of iopamidol (delta Hounsfield units; *Δ*HU) and representative CT images (axial view) of **K**PC961 orthotopic tumors (red contour) before (0 min) and up to 40 min following iopamidol injection by IP; (b) quantification of iopamidol (*Δ*HU) and representative CT images (axial view) of Panc02 orthotopic tumors (red contour) before (0 min) and 40 min postiopamidol administration by IP. Hounsfield units (HU).

Results showed that IV plus infusion protocol provided a continuous uptake increase of iopamidol into the KPC961 tumors, presenting *Δ*HU values from 44.54 ± 9.36 to 63.33 ± 8.37, 15 to 25 min postinjection (Figures 2(a) and 2(b)). IP bolus injection was also effective in delivering similarly high amounts of iopamidol into KPC961 orthotopic tumor, showing *Δ*HU values ranging from 42.2 ± 9.32 to 56.09 ± 11.97, from 30 to 40 min postinjection (Figure 3(a)). Similarly, Panc02 orthotopic tumors presented *Δ*HU values of 43.08 ± 12.58 at 40 min post-IP injection (Figure 3(b)). To avoid streak artifacts due to the iopamidol bolus present in the abdomen, we did not quantify the iopamidol concentration inside the pancreatic tumors for the first 10 min when using the IP injection protocol.

**Figure 4:**
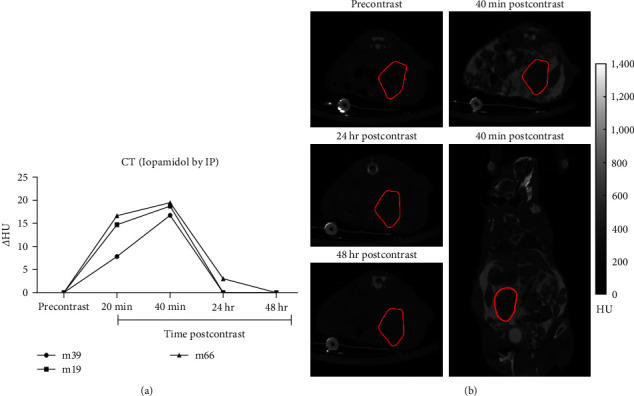
Clearance of iopamidol in KPC961 orthotopic tumors following IP injection protocol. (a) Delta Hounsfield units (*Δ*HU) were obtained by subtracting the mean intensity from the tumor ROI in each postcontrast CT image (20, 40 min, 24, and 48 hr) from the mean intensity of the tumor ROI in the precontrast CT image; (b) representative CT images showing tumor ROI in red and axial view for the precontrast, axial, and coronal views for the 40 min postcontrast, and axial views for 24 and 48 hr postcontrast CT images. Hounsfield units (HU).

In addition, we evaluated the clearance of iopamidol in KPC961 orthotopic tumors following the IP injection protocol. Mice were imaged before contrast injection, and at 20, 40 min, 24, and 48 hr postinjection. Results showed that after 24 hr there was minimal (*Δ*HU = 3.03) or no iopamidol inside the tumors, and it was completely cleared 48 hr post-IP injection (Figure 4). This is especially important for designing longitudinal studies, where the contrast agent can be reinjected every 48 hr.

### 3.2. pHe Measurements Obtained by CEST-MRI

Next, we used CEST-MRI to test if iopamidol delivered by IP would provide accurate tumor pHe maps in KPC961 orthotopic model. Since IV is the most common route used for administration of contrast agents, we compared the pHe maps obtained when iopamidol was administered by IP with pHe maps obtained when iopamidol was injected using an IV plus infusion protocol.

**Figure 5:**
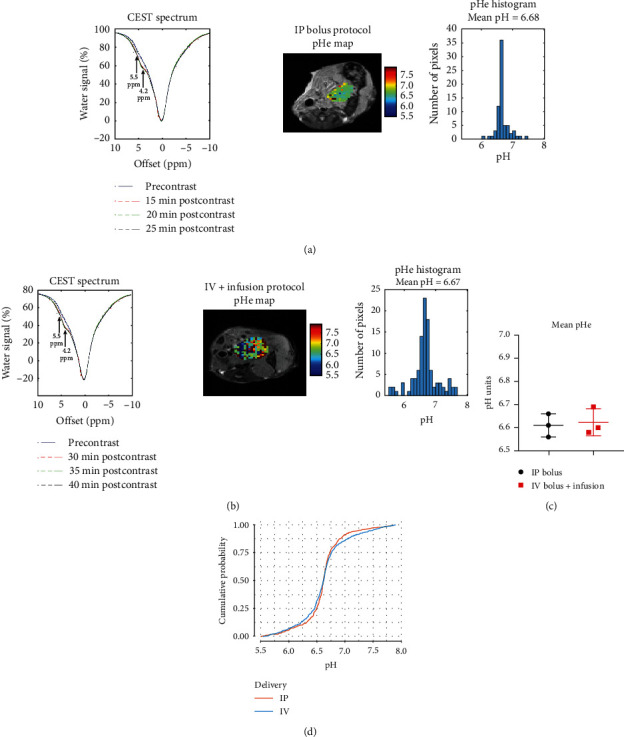
Assessment of tumor pHe by CEST-MRI with iopamidol delivered by IP (A) or IV (B) routes in KPC961 orthotopic tumors. (a) Representative CEST spectra showing CEST effects at 4.2 and 5.5 ppm, pHe maps, and histograms showing pHe pixel distributions in KPC961 tumors obtained when iopamidol was delivered by IP bolus injection, and (b) when using the IV + infusion injection protocol. CEST spectra are shown for the regions of interest (ROI), which was drawn around the tumor. Dots represent the raw data, dashed, and solid curves represent the precontrast and postcontrast least-squares fitting of the data to the multipool lorentzian model. Mean pHe values were calculated by averaging the pHe values obtained in the last three postcontrast time points for each injection protocol, but the pHe maps shown are the most representative of one of the postcontrast time points used to calculate the mean pHe; (c) comparison of mean pHe values between iopamidol injection protocol delivered by IP or IV using Student's *t*-test. No statistical difference was observed (*p*=0.77); (d) empirical cumulative density functions (ECDF) of pHe pixel distribution in the tumors and statistical comparison using Kolmogorov–Smirnov test. No statistical significance was observed between IP and IV iopamidol injection protocols (*p*=0.16).

Figures 5(a) and 5(b) show a representative CEST spectrum, tumor pHe map, and histograms with pHe pixel distribution for each injection protocol. As expected, both IP and IV injection protocols produced comparable measurements of tumor pHe, showing no statistically significant difference when comparing the mean tumor pHe between groups (*p*=0.77; Figure 5(c)). This was also confirmed in further statistical analysis, using ECDF curves coupled with Kolmogorov–Smirnov test. This analysis allows us to examine and compare the distributions of pHe throughout all the voxels of the tumor, which showed no significantly statistical differences between groups (*D* = 0.06, *p*=0.16; Figure 5(d)).

### 3.3. Evaluating the Effect of Bicarbonate on Tumor pHe using CEST-MRI

To test the ability to conduct longitudinal pHe measurements in orthotopic pancreatic tumors using CEST-MRI with iopamidol delivered by IP, we evaluated changes in tumor pHe following bicarbonate administration.

**Figure 6:**
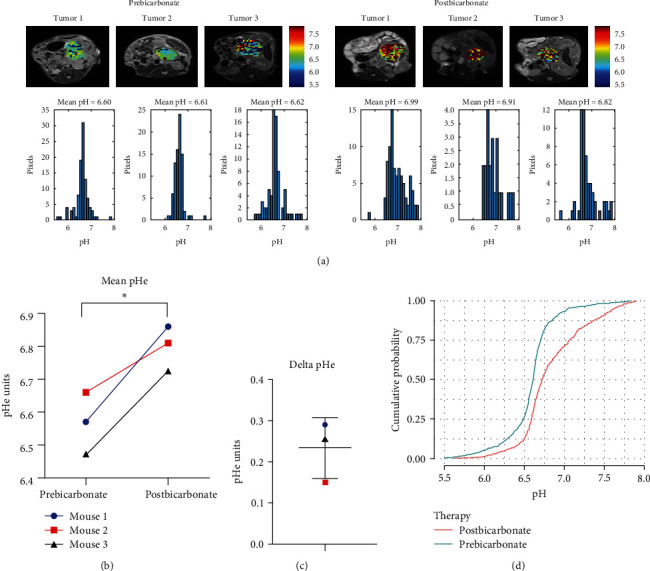
Longitudinal changes in tumor pHe obtained by CEST-MRI using iopamidol delivered by IP in response to bicarbonate treatment in orthotopic pancreatic tumors. (a) Representative pHe maps and histograms of pHe pixel distributions in KPC961 clone 1B6 orthotopic tumors at baseline (prebicarbonate) and following bicarbonate administration by gavage 2 days later; (b) comparison of tumor pHe mean values showing a statistically significant increase in response to bicarbonate treatment; (c) delta pHe obtained by subtracting the postbicarbonate tumor pHe from baseline tumor pHe for each mouse; (d) empirical cumulative density functions (ECDF) of pHe pixel distribution in the tumors and statistical comparison using Kolmogorov–Smirnov test. The ECDF curves differ significantly with postbicarbonate showing higher pHe values.

Results showed an increase in tumor pHe following bicarbonate administration for all three mice tested (Figure 6(a)–6(c)). Baseline mean pHe values for each tumor were 6.57, 6.66, and 6.47 and they increased to 6.86, 6.81, and 6.72 after bicarbonate, respectively. This increase was significantly different when comparing pre- and postbicarbonate mean tumor pHe values using paired Student *t*-test (*p*=0.03; Figure 6(b)). ECDFs coupled with Kolmogorov–Smirnov test confirmed the increase in tumor pHe after bicarbonate (*D* = 0.29; *p*=2.2e − 16; Figure 6(d)). Figure 6(d) shows clearly that baseline pHe (prebicarbonate gavage) lies entirely to the leFt of the pH axis compared to that of postbicarbonate gavage, indicating that it was more acidic throughout the tumor ROI prebicarbonate compared to postbicarbonate treatment. Moreover, the lowest pHe for tumors at baseline is more acidic than the lowest pHe for tumor postbicarbonate treatment, and the highest pHe (least acidic) for tumors postbicarbonate is greater than the least acidic pHe for tumors at baseline. Further, almost 90% of the pixels in the tumors at baseline have a pHe < 7, whereas only about 70% of the pixels for tumors postbicarbonate have a pHe < 7. These results demonstrate that the bicarbonate gavage decreased acidity throughout the entire tumor ROI.

## 4. Discussion

In the present study, we demonstrated the feasibility of monitoring longitudinal tumor pHe using CEST-MRI with iopamidol delivered by IP injection in orthotopic pancreatic tumors. The time-series CT showed that 40 min after IP bolus injection of iopamidol, tumor iopamidol uptake was similar to that achieved 25 min postinjection using a standard IV plus infusion protocol. Likewise, for both injection protocols, the tumor uptake of iopamidol was satisfactory, reaching concentrations that were sufficient to generate reliable CEST signal detection, and resulting in comparable pixel-wise pHe maps between IV and IP protocols. In addition, we showed that within 48 hr of iopamidol injection by IP, it was completely cleared from orthotopic tumors, demonstrating the ability to conduct CEST longitudinal imaging. Finally, CEST-MRI with IP delivery of iopamidol was able to detect changes in tumor pHe in response to bicarbonate administration. Therefore, pH responsiveness of iopamidol-based tumor pH imaging is not dependent on administration protocols, since other studies demonstrated increased tumor pH upon bicarbonate administration following IV delivery of iopamidol [20, 27].

Different routes of administration for a contrast agent for CEST-MRI (iopromide; 300 mg iodine/ml) were evaluated in another study with a breast cancer subcutaneous model [14]. In contrast with our orthotopic pancreatic model, those authors showed that an IP injection (1.5 ml) of iopromide showed no detectable uptake in the subcutaneous breast tumor, and limited uptake in bladder and kidneys [14]. We can hypothesize that the contrasting results between our study and theirs may be due to biological differences in the tumor models, particularly with respect to the tumor site. Orthotopic tumors exhibit higher vascular perfusion compared to subcutaneous tumors [28], which may impact the rate of contrast uptake by orthotopic and subcutaneous tumors. Although higher percent uptake of the agent during CEST-MRI would suggest higher vascular permeability and greater angiogenesis, it should be used only as a qualitative assessment rather than quantitative [9]. Ensuring accumulation of sufficient concentration of the contrast agent throughout the entire extracellular microenvironment is important to eliminate bias, where tumor regions with relatively poor vascular perfusion, such as the tumor core can be misrepresented, with the mean tumor pHe biased toward tumor regions that present high vascular perfusion and permeability [21]. Moreover, the IV protocol provided a prolonged accumulation of iopamidol up to 25 min postinjection, as measured with the CT technique, as previously observed upon IV administration of several iodinated contrast media in a subcutaneous HER2+ breast cancer murine model [29].

Other studies implemented different MRI techniques to measure tissue pH, such as MR spectroscopy (MRS) [30, 31] and hyperpolarization [32]. MRS-pH imaging relies on the presence of two peaks, ideally pH-dependent and pH-independent, and their chemical shift difference to exploit the ratiometric approach to extrapolate pH. Notably, ^31^P MRS studies reported fundamental results about tumor acidosis both at intracellular and extracellular levels [33, 34], showing the pH gradient between the two tumor compartments and their differences compared to the normal tissue [35]. On the other hand, hyperpolarized MRI uses polarization transfer from electrons to nuclei to strongly increase the sensitivity for MRS. ^13^C-labeled molecules are the most used with this technique [36] and pH estimations can be achieved by designing molecules with pH-dependent chemical shift or, in the case of hyperpolarized ^13^C bicarbonate, the ratio of signal concentrations calculated through the signal intensity changes between H^13^CO_3_^−^ and ^13^CO_2_ can be used to measure pH using the Henderson−Hasselbalch equation [37]. The potential of the approach has been explored in many studies at both preclinical and clinical levels [38, 39] revealing pH measurements in good agreement with those obtained with other techniques [40, 41]. Although the promising results arose from studies on tumor metabolism and response to therapy [24, 42–44] the two techniques have some limitations mainly due to poor sensitivity, challenging pH-sensor development, elaborated equipment, and low spatial resolution yielding to difficult application *in vivo* and in clinical settings.

CEST-MRI studies have shown the feasibility of performing pH measurements coupled with high spatial resolution and good sensitivity, allowing the monitoring of early response to therapies [21] and thus, the detection of therapy response and the onset of resistance [45]. Furthermore, the CEST-pH approach was able to detect changes in tumor pHe in a mammary tumor model (4T1) following treatment with a mitochondrial pyruvate carrier (MPC) inhibitor (UK-5099), where ^31^P NMR spectroscopy did not detect any significant changes. In particular, analysis of histograms of distributions of pHe values revealed that a small part of the tumors was affected by extracellular acidification [46]. In addition to response studies, improvements in imaging acquisitions and volume coverage were achieved with multislice CEST acquisitions allowing a more comprehensive and extensive visualization of tumor extracellular pH [47–50].

Although we have demonstrated that contrast agent IP injection is feasible for preclinical imaging, there is also a translational potential for the use of IP in the clinical practice. In particular, CT peritoneography is performed using the peritoneal dialysis (PD) catheter to infuse a diluted solution of the contrast agent mixed with the dialysate. Generally, this approach is used to verify abdominal wall integrity and to see if there could be any leakage or local accumulation of dialysate during the PD procedure. A great advantage of this injection technique is that after the CT is performed, the excess of contrast agent can be promptly removed once the dialysis process is restored [51, 52].

In addition, a particular advantage of our protocol in the preclinical setting is that delivering iopamidol IP can increase the number of CEST-MRI sessions that each mouse can undergo for longitudinal pHe measurements, always accounting for the limits imposed by animal welfare and study termination rules. For experiments where a contrast agent is delivered IV, most animals can be imaged three times until repeated tail vein catheterizations reduce the tissue integrity of the tail, limiting the number of imaging sessions [21]. With IP delivery, the tail is preserved for repeated drug treatments allowing an increased success of IV injections throughout the study. However, CEST techniques are not exempt from challenges that could limit their applicability. It is important to acknowledge that the use of IP injection requires a longer acquisition time, adding an extra 15–20 min in the protocol compared with the IV injection. Long acquisition times, concern related to specific absorption rate (SAR) due to the radiofrequency power used, B_1_ and B_0_ inhomogeneities and motion artifacts are the main vulnerabilities, but they can be addressed with specific sequence designs and postprocessing analyses [10, 53, 54].

## 5. Conclusion

This study shows the applicability of the IP delivery of iopamidol to conduct longitudinal CEST-pH measurements in pancreatic tumor models, as demonstrated with contrast agent quantification in CT imaging and with MRI-pH imaging experiments.

## Data Availability

The data that support the findings of this study are available from the corresponding author.

## Supplementary Materials

Supplementary Materials Figure S1: *In vitro* calibration curve for pH determination.

## References

[1] Lindeman L. R., Randtke E. A., High R. A., Jones K. M., Howison C. M., Pagel M. D. (2018). A comparison of exogenous and endogenous CEST MRI methods for evaluating in vivo pH. *Magnetic Resonance in Medicine*.

[2] Damaghi M., Wojtkowiak J. W., Gillies R. J. (2013). pH sensing and regulation in cancer. *Frontiers in Physiology*.

[3] Wojtkowiak J. W., Verduzco D., Schramm K. J., Gillies R. J. (2011). Drug resistance and cellular adaptation to tumor acidic pH microenvironment. *Molecular Pharmaceutics*.

[4] Ibrahim-Hashim A., Estrella V. (2019). Acidosis and cancer: from mechanism to neutralization. *Cancer and Metastasis Reviews*.

[5] Robey I. F., Baggett B. K., Kirkpatrick N. D. (2009). Bicarbonate increases tumor pH and inhibits spontaneous metastases. *Cancer Research*.

[6] Chen L. Q., Pagel M. D. (2015). Evaluating pH in the extracellular tumor microenvironment using CEST MRI and other imaging methods. *Advances in Radiology*.

[7] Hyder F., Coman D. (2021). Imaging extracellular acidification and immune activation in cancer. *Current Opinion in Biomedical Engineering*.

[8] Anemone A., Consolino L., Arena F., Capozza M., Longo D. L. (2019). Imaging tumor acidosis: a survey of the available techniques for mapping *in vivo* tumor pH. *Cancer and Metastasis Reviews*.

[9] Chen L. Q., Randtke E. A., Jones K. M., Moon B. F., Howison C. M., Pagel M. D. (2015). Evaluations of tumor acidosis within *in vivo* tumor models using parametric maps generated with acidoCEST MRI. *Molecular Imaging and Biology*.

[10] Vinogradov E., Sherry A. D., Lenkinski R. E. (2013). CEST: from basic principles to applications, challenges and opportunities. *Journal of Magnetic Resonance*.

[11] van Zijl P. C. M., Yadav N. N. (2011). Chemical exchange saturation transfer (CEST): what is in a name and what isn’t?. *Magnetic Resonance in Medicine*.

[12] Chen Z., Han Z., Liu G. (2021). Repurposing clinical agents for chemical exchange saturation transfer magnetic resonance imaging: current status and future perspectives. *Pharmaceuticals*.

[13] Sun P. Z., Longo D. L., Hu W., Xiao G., Wu R. (2014). Quantification of iopamidol multi-site chemical exchange properties for ratiometric chemical exchange saturation transfer (CEST) imaging of pH. *Physics in Medicine & Biology*.

[14] Chen L. Q., Howison C. M., Jeffery J. J., Robey I. F., Kuo P. H., Page M. D. (2014). Evaluations of extracellular pH within in vivo tumors using acidoCEST MRI. *Magnetic Resonance in Medicine*.

[15] Longo D. L., Dastrù W., Digilio G. (2011). Iopamidol as a responsive MRI-chemical exchange saturation transfer contrast agent for pH mapping of kidneys: in vivo studies in mice at 7 T. *Magnetic Resonance in Medicine*.

[16] Longo D. L., Sun P. Z., Consolino L., Michelotti F. C., Uggeri F., Aime S. (2014). A general MRI-CEST ratiometric approach for pH imaging: demonstration of *in vivo* pH mapping with iobitridol. *Journal of the American Chemical Society*.

[17] Jones K. M., Pollard A. C., Pagel M. D. (2018). Clinical applications of chemical exchange saturation transfer (CEST) MRI. *Journal of Magnetic Resonance Imaging*.

[18] Tang Y., Xiao G., Shen Z. (2020). Noninvasive detection of extracellular pH in human benign and malignant liver tumors using CEST MRI. *Frontiers in Oncology*.

[19] High R. A., Ji Y., Ma Y.-J. (2019). *In vivo* assessment of extracellular pH of joint tissues using acidoCEST-UTE MRI. *Quantitative Imaging in Medicine and Surgery*.

[20] Longo D. L., Bartoli A., Consolino L. (2016). *In vivo* imaging of tumor metabolism and acidosis by combining PET and MRI-CEST pH imaging. *Cancer Research*.

[21] Akhenblit P. J., Hanke N. T., Gill A. (2016). Assessing metabolic changes in response to mTOR inhibition in a mantle cell lymphoma xenograft model using acidoCEST MRI. *Molecular Imaging*.

[22] Ibrahim-Hashim A., Robertson-Tessi M., Enriquez-Navas P. M. (2017). Defining cancer subpopulations by adaptive strategies rather than molecular properties provides novel insights into intratumoral evolution. *Cancer Research*.

[23] Pilon-Thomas S., Kodumudi K. N., El-Kenawi A. E. (2016). Neutralization of tumor acidity improves antitumor responses to immunotherapy. *Cancer Research*.

[24] Raghunand N., Mahoney B., Sluis R., Baggett B., Gillies R. J. (2001). Acute metabolic alkalosis enhances response of C3H mouse mammary tumors to the weak base mitoxantrone. *Neoplasia*.

[25] Wu Y., Zhou I. Y., Igarashi T., Longo D. L., Aime S., Sun P. Z. (2018). A generalized ratiometric chemical exchange saturation transfer (CEST) MRI approach for mapping renal pH using iopamidol. *Magnetic Resonance in Medicine*.

[26] Team R. C. (2020). R: A language and environment for statistical computing.

[27] Anemone A., Consolino L., Conti L. (2021). Tumour acidosis evaluated in vivo by MRI-CEST pH imaging reveals breast cancer metastatic potential. *British Journal of Cancer*.

[28] Zhang W., Fan W., Rachagani S. (2019). Comparative study of subcutaneous and orthotopic mouse models of prostate cancer: vascular perfusion, vasculature density, hypoxic burden and BB2r-targeting efficacy. *Scientific Reports*.

[29] Longo D. L., Michelotti F., Consolino L. (2016). In vitro and in vivo assessment of nonionic iodinated radiographic molecules as chemical exchange saturation transfer magnetic resonance imaging tumor perfusion agents. *Investigative Radiology*.

[30] Ren J., Sherry A. D., Malloy C. R. (2015). ^31^P-MRS of healthy human brain: ATP synthesis, metabolite concentrations, pH, and T_1_ relaxation times. *NMR in Biomedicine*.

[31] Kintner D. B., Anderson M. K., Fitzpatrick J. H., Sailor K. A., Gilboe D. D. (2000). 31P-MRS-based determination of brain intracellular and interstitial pH: its application to in vivo H+ compartmentation and cellular regulation during hypoxic/ischemic conditions. *Neurochemical Research*.

[32] Gallagher F. A., Kettunen M. I., Brindle K. M. (2011). Imaging pH with hyperpolarized ^13^C. *NMR in Biomedicine*.

[33] Gillies R. J., Liu Z., Bhujwalla Z. (1994). 31P-MRS measurements of extracellular pH of tumors using 3-aminopropylphosphonate. *American Journal of Physiology-Cell Physiology*.

[34] Lutz N. W., Fur Y. L., Chiche J., Pouysségur J., Cozzone P. J. (2013). Quantitative *in vivo* characterization of intracellular and extracellular pH profiles in heterogeneous tumors: a novel method enabling multiparametric pH analysis. *Cancer Research*.

[35] Gerweck L. E., Seetharaman K. (1996). Cellular pH gradient in tumor versus normal tissue: potential exploitation for the treatment of cancer. *Cancer Research*.

[36] Nelson S. J., Vigneron D., Kurhanewicz J., Chen A., Bok R., Hurd R. (2008). DNP-hyperpolarized ^13^C magnetic resonance metabolic imaging for cancer applications. *Applied Magnetic Resonance*.

[37] Gallagher F. A., Kettunen M. I., Day S. E. (2008). Magnetic resonance imaging of pH *in vivo* using hyperpolarized ^13^C-labelled bicarbonate. *Nature*.

[38] Serrao E. M., Brindle K. M. (2016). Potential clinical roles for metabolic imaging with hyperpolarized [1-^13^C]pyruvate. *Frontiers in Oncology*.

[39] Lim H., Albatany M., Martínez-Santiesteban F., Bartha R., Scholl T. J. (2018). Longitudinal measurements of intra- and extracellular pH gradient in a rat model of glioma. *Tomography*.

[40] Düwel S., Hundshammer C., Gersch M. (2017). Imaging of pH *in vivo* using hyperpolarized 13C-labelled zymonic acid. *Nature Communications*.

[41] Hundshammer C., Düwel S., Köcher S. S. (2017). Deuteration of hyperpolarized ^13^C-labeled zymonic acid enables sensitivity-enhanced dynamic MRI of pH. *ChemPhysChem*.

[42] Cavallari E., Carrera C., Aime S., Reineri F. (2019). Metabolic studies of tumor cells using [1-^13^C] pyruvate hyperpolarized by means of PHIP-side arm hydrogenation. *Chemphyschem*.

[43] Raghunand N., He X., van Sluis R. (1999). Enhancement of chemotherapy by manipulation of tumour pH. *British Journal of Cancer*.

[44] Ibrahim Hashim A., Cornnell H. H., Coelho Ribeiro M. D. L. (2011). Reduction of metastasis using a non-volatile buffer. *Clinical & Experimental Metastasis*.

[45] Anemone A., Consolino L., Conti L. (2017). In vivo evaluation of tumour acidosis for assessing the early metabolic response and onset of resistance to dichloroacetate by using magnetic resonance pH imaging. *International Journal of Oncology*.

[46] Buyse C., Joudiou N., Corbet C. (2021). Impact of inhibition of the mitochondrial pyruvate carrier on the tumor extracellular pH as measured by CEST-MRI. *Cancers*.

[47] Sun P. Z., Cheung J. S., Wang E., Benner T., Sorensen A. G. (2011). Fast multislice pH-weighted chemical exchange saturation transfer (CEST) MRI with unevenly segmented RF irradiation. *Magnetic Resonance in Medicine*.

[48] Kim H., Krishnamurthy L. C., Sun P. Z. (2022). Demonstration of fast multi-slice quasi-steady-state chemical exchange saturation transfer (QUASS CEST) human brain imaging at 3T. *Magnetic Resonance in Medicine*.

[49] Villano D., Romdhane F., Irrera P. (2021). A fast multislice sequence for 3D MRI-CEST pH imaging. *Magnetic Resonance in Medicine*.

[50] Randtke E. A., Granados J. C., Howison C. M., Pagel M. D., Cárdenas-Rodríguez J. (2017). Multislice CEST MRI improves the spatial assessment of tumor pH. *Magnetic Resonance in Medicine*.

[51] Karahan O. I., Taşkapan H., Tokgöz B., Coşkun A., Utaş C., Güleç M. (2002). Continuous ambulatory peritoneal dialysis: CT peritoneography findings and assessment of related clinical complications. *Acta Radiologica*.

[52] Rappai J., Crabtree J. H., Mancini A., Badugu S. K., Kaushal A., Gellens M. E. (2022). Compatibility and stability of non-ionic iodinated contrast media in peritoneal dialysis solution and safe practice considerations for CT peritoneography. *Peritoneal Dialysis International: Journal of the International Society for Peritoneal Dialysis*.

[53] Huang J., Chen Z., Park S.-W., Lai J. H. C., Chan K. W. Y. (2022). Molecular imaging of brain tumors and drug delivery using CEST MRI: promises and challenges. *Pharmaceutics*.

[54] Romdhane F., Villano D., Irrera P., Consolino L., Longo D. L. (2021). Evaluation of a similarity anisotropic diffusion denoising approach for improving in vivo CEST-MRI tumor pH imaging. *Magnetic Resonance in Medicine*.

