# Supplementary material for: Intraperitoneal Delivery of Iopamidol to Assess Extracellular pH of Orthotopic Pancreatic Tumor Model by CEST-MRI
Source: Contrast Media Mol Imaging. 2023 Jan 5;2023:1944970. doi: 10.1155/2023/1944970 (PMC9836819; doi:10.1155/2023/1944970)
Supplement: Supplementary Materials — Figure S1: In vitro calibration curve for pH determination. [file 1944970.f1.docx]

**Supplementary Figure 1.** *In vitro* calibration curve for pH determination. Four separate MR acquisitions were performed with a phantom consisting of 7 vials at different pH values (5.75 to 7.75) of a 20 mM iopamidol solution in 1X PBS. The log10 ratio between the iopamidol frequencies was used after deriving the signal intensity values with a 3-pools Lorentzian fitting. A polynomial function of degree 3 was then used to fit the experimental data.
